# In what ways does the mandatory nature of Victoria's municipal public health planning framework impact on the planning process and outcomes?

**DOI:** 10.1186/1743-8462-4-4

**Published:** 2007-03-22

**Authors:** Prue Bagley, Vivian Lin, Peter Sainsbury, Marilyn Wise, Tom Keating, Karen Roger

**Affiliations:** 1 La Trobe University, Melbourne, Australia; 2 Sydney South West Area Health Service, Sydney, Australia; 3 University of Sydney, Sydney, Australia; 4 Jesuit Social Services, Australia; 5 National Public Health Partnership, Melbourne, Australia

## Abstract

**Background:**

Systems for planning are a critical component of the infrastructure for public health. Both in Australia and internationally there is growing interest in how planning processes might best be strengthened to improve health outcomes for communities. In Australia the delivery of public health varies across states, and mandated municipal public health planning is being introduced or considered in a number of jurisdictions. In 1988 the Victorian State government enacted legislation that made it mandatory for each local government to produce a Municipal Public Health Plan, offering us a 20-year experience to consider.

**Results:**

In-depth interviews were undertaken with those involved in public health planning at the local government level, as part of a larger study on local public health infrastructure and capacity. From these interviews four significant themes emerge. Firstly, there is general agreement that the Victorian framework of mandatory public health planning has led to improvements in systems for planning. However, there is some debate about the degree of that improvement. Secondly, there is considerable variation in the way in which councils approach planning and the priority they attach to the process. Thirdly, there is concern that the focus is on producing a plan rather than on implementing the plan. Finally, some tension over priorities is evident. Those responsible for developing Municipal Public Health Plans express frustration over the difficulty of having issues they believe are important addressed through the MPHP process.

**Conclusion:**

There are criticisms of Victoria's system for public health planning at the local government level. Some of these issues may be specific to the arrangement in Victoria, others are problems encountered in public health planning generally. In Victoria where the delivery structure for public health is diverse, a system of mandatory planning has created a minimum standard. The implementation of the framework was slow and factors in the broader political environment had a significant impact. Work done in recent years to support the process appears to have led to improvements. There are lessons for other states as they embark upon mandated public health plans.

## Background

Strengthening public health infrastructure and capacity is an issue attracting increasing national and international interest (see for example [1-4] and [5]). In the USA and UK, attention has turned to strengthening public health systems for emergency preparedness following September 2001, the SARs outbreak and, more recently, avian influenza. One component of this work has been a focus on how to improve planning processes at the community level (see for example [6,7] and [8]). In Australia, interest is also driven by the increasing complexity of the issues faced by public health, shrinking resources and 'reforms' in public administration. Although infrastructure and capacity have been variously defined, systems for planning are generally considered to be a critical component (for definitions of infrastructure see [1,9-11] and [12]). Australian activities in formal health planning date from the 1970s during which time there have been paradigmatic shifts, from an interest in equity to a focus on efficiency, that mirror changes in the broader policy environment [13] (see pages 55–67). Currently, a central concern is how planning processes might be strengthened to improve outcomes in the public's health.

In Australia the configuration of the delivery system for public health varies from state to state. In Victoria much of its public health services are delivered by local governments (see [14] for an extensive review of the role played by local government across Australia). The 79 Victorian local governments vary in a number of ways including *inter alia *size, geography, population, and organisational income and capacity. They are an autonomous and separate sphere of government, although a great deal of what they do is prescribed in legislation set by State government. In addition, funding agreements with Federal and State agencies dictate a number of their activities. In 1988 the State government amended the 1958 Health Act and made it mandatory for each local government (council) to develop a municipal public health plan (MPHP). The Health (General Amendment) Act 1988 (29B) states that:

"A municipal public health plan must: (a) identify and assess actual and potential public health dangers affecting the municipal district; and (b) outline programs and strategies which the council intends to pursue to (i) prevent or minimise those dangers; and (ii) enable people living in the municipal district to achieve maximum well-being; and (c) provide for periodic evaluation of programs and strategies."

Councils are required to prepare a new plan every three years and review their existing plan annually. These provisions, within the legislation, constitute Victoria's municipal public health planning framework.

MPHPs are not the only legislated plans at the local level in Victoria. The Local Government Act 1989 and the Planning and Environment Act 1987 both impose planning requirements on councils. In addition to these plans there are numerous other plans required and discussions are underway between State and local government about how the administrative workload they impose might best be reduced [15].

Another review of the Health Act was initiated in 2002, and some of the issues highlighted for comment relate directly to MPHP activities. This review makes discussion of the planning framework's impact to date particularly timely. However, it is not simply an issue for Victoria. The Western Australian government is reviewing its Health Act and asking "should the public health powers, functions and responsibilities of local government be spelt out in a new Health Act in more detail than currently exists?" [16] (see page 14). All jurisdictions have an interest in how best to plan for and deliver public health services.

As a system for planning, the framework in Victoria is unique in that it is mandatory. The aim of this article is to review the strengths and weaknesses of this framework, as it was implemented in Victoria. Of particular interest are the questions: has the Victorian framework improved planning processes and outcomes; what has been the extent of any improvement; and what are the challenges that remain? Underpinning these questions is the broader issue regarding the degree to which problems, where identified, are specific to this Victorian framework for planning or are general problems encountered in public health planning, irrespective of the manner in which it is organised.

## Results

Analysis of the interview transcripts provided a great deal of information about the planning process from which four significant, and interrelated, themes emerge: agreement that the legislation has improved systems for planning but debate about the extent of that improvement; significant variation in the way in which councils approach planning, their level of sophistication and the priority they attach to the process; concern that the focus is primarily on the process itself (producing a plan) rather than on outcomes (implementing the plan); and some tension over priorities, with frustration expressed by those responsible for developing MPHPs that areas they believed should have priority are either not included in plans or where they are included they may be relegated in importance when State and Federal government prioritise other activities.

### Legislation has improved planning

The majority of those interviewed believed that, on balance, the legislative mandate had improved the planning process. Local government representatives expressed a variety of reactions to the MPHP process. As the following quotes illustrate, some saw it as a useful activity that helped in 'making a case for public health' within the broader council environment and increased the visibility of public health, whilst for others it was simply another function imposed by State government:

"they [council] have come around to understanding the importance of a public health plan and have, quite rightly, given it the resourcing it requires."

"the way we structured it [the MPHP] is to try and take the role of integrating public health thinking across the council's strategic planning process, and we are trying to lift awareness across council"

"it is not something that is high priority. Most councils don't prioritise it terribly highly ... it has to be reproduced every three years and it has to be reviewed every 12 months but in practice most people just produce it every three years and forget about it"

Those outside local government were generally, but not exclusively, more positive about the MPHP process and outcomes. They believed that publications by the Department of Human Services [17] and the Victorian Health Promotion Foundation [18] had strengthened local government planning functions. One respondent identified what he believed to be a genuine shift in thinking about planning away from what he classified as 'retrospective planning', where councils simply developed a plan that reported what they had done in the past, towards more strategic planning. Despite general agreement that the system had improved the planning process, there were concerns about the quality of planning:

"it just depends on the council and how advanced they are. Some, like [name of council] are very good and you don't have any problem with them but there are others who ... they just don't get it basically"

### Significant variation exists

Significant variations exist in the way in which councils approach the MPHP process. This was evident from the manner in which those within local government talked about the process, as the two following quotes illustrate:

"State government sets a number of key performance indicators that we pick up on and sort of blend our own stuff into"

"we ended up with a really energised, mobilised community [as a result of the planning process], who not only saw their name or their idea in the plan but who were also really motivated to do something about it"

Comments from those external to local government also corroborate the significant variations at play:

"we have a bit of a spectrum from those who have got a real community development, community participation focus and a real strong social model of health focus to those who have taken the National Health Priority Areas and modelled their health planning around health issues and diseases and risk factors"

"the ones that I've seen that have more of a focus in community, that really have got their community strategies right, are really doing some good work"

### The focus is primarily on the process itself rather than on outcomes

Another central concern raised was the extent to which legislation emphasised the process of planning but not the outcomes:

"because notoriously in the past many local governments ... even though they have a statutory obligation to produce a plan ... they would produce it and it might just end up on the bookshelf"

"I was going to say outcomes but you know there are no outcomes to some of their municipal public health planning endeavours, you know apart from the obvious immunisation stuff that they have done forever anyway, I mean some of them are lucky if they can throw together a document"

All the councils interviewed had an MPHP but about half lacked a formal implementation strategy.

### Tension over priorities

Within council there can be resistance to including anything within a plan that senior management and/or councillors do not see as the 'core business' of council. One respondent noted that his community health unit had identified the need for low cost housing in the municipality but his council certainly did not see itself in the business of providing housing. Another noted that:

"even though you might identify something as an issue there is no guarantee that it is going to be addressed in any proactive way"

Even if health planners are successful in having an issue included in the plan, this does not guarantee that it will be addressed. The process may then be overtaken by political demands, either from Federal or State government or from within council itself, usually as a response to community or political pressure. Immunisation and illicit drugs are two examples of this. In the first instance, Federal government recently decided to increase the age range for children being immunised. This resulted in significant extra work in the states. In Victoria a great deal of this increase in work was borne by public health units within local government. As funding is contingent on this being undertaken, other activities within the MPHP are relegated:

"meningococcal immunisation was a case in point. The Federal government decided it was something that they should fund for all children from the age of birth to 20 years ... well who's going to do it? There is a statutory requirement on local government to do immunisation. So that meant that instead of doing our normal workload for immunisation, the workload tripled simply because the number of kids tripled"

In the case of illicit drugs, an apparent spike in the number of deaths, particularly in public spaces, resulted in a great deal of media coverage and significant community concern. Funding became available and programs were developed in response to the newly presented funding opportunities. As one of the respondents noted:

"the previous municipal public health plan did identify a need for a drug policy of some sort or some attention on drugs which we did achieve, but not directly through the municipal public health plan. It was an adjunct of people dying on the streets"

A number of participants observed that everything 'comes down to money'. While most of those interviewed spoke about this phenomena in negative terms, as peripatetic crisis funding, one person observed that a strong public health unit could and should respond to these opportunities:

"we are looking to move ... towards a much more complex and much more flexible, much more flexible arrangement so that we can become, for want of a better word, 'nippy' you know so that we can nip in and out where things happen and where we can make an impact"

## Discussion

The four major themes that emerge from the interviews (improvement in planning systems; variation in approaches to planning; an emphasis on the process rather than on outcomes; and tensions over priorities) indicate something of the strengths and weaknesses of the Victorian planning framework. It is worthwhile to look at the intent of the legislation, its implementation and the value of the MPHP framework.

### The intent

Local government in Australia has played a role in public health since the 19^th ^century and, as Legge and Cox argue, "should be acknowledged as one of the main institutional foundations of public health practice" [19] (see page 3). Their initial, and in many cases continuing, role centred on traditional public health issues such as sanitation and waste management. In the 20^th ^century the role of many local governments began to expand beyond an interest in the physical environment [20]. The pace of this expansion increased from the mid-century. It occurred at a differential rate from state to state and was most notable in Victoria [14] (see page 100). By the 1970s Victoria's local governments had a role in maternal and child health, childhood immunisation programs, services for older people and more general welfare services [20,19]. As Smith notes, one of the significant features of this expansion was a growing interest in "people and their social circumstances rather than on the physical environment" [20] (see page 6).

In the mid-1980s the Victorian state government undertook a series of reviews of health legislation, including the Health Act 1958 that defines the roles and responsibilities of local government as they relate to public health. Smith argues that there are two salient points to note about the intent underpinning the review of the Health Act: that it "regarded public health in terms of the New Public Health" and that it "would involve the examination of the relationship between the then Health Department Victoria and local government" [20] (see page 7). However, as both Smith [20] and Wills [21] note, despite using the language of health promotion, the emphasis in the relevant sections of the legislation (29A and 29B) was on protection and prevention – the so called 'old' public health. Wills [21] argues that this disjunction led to confusion about the scope of the MPHPs.

The Act was an attempt to introduce positive, rather than punitive, legislation that would encourage councils to act in a certain way rather than punish them for failing to do so. In the discussion document preceding the legislation the Health Department noted that:

"Health Department Victoria (HDV) must be careful not to impose rigid regulatory controls to be enforced by councils and which limit their ability to re-determine their own priorities in response to changing public health needs in the municipality" [22] (see page 11).

This notion of positive legislation is reinforced by the language used in Department of Human Services (DHS) supporting documents where the Act is represented as a 'planning framework' rather than a 'planning template'. The choice of language is deliberate, the distinction being that frameworks allow for a bottom-up approach where templates reflect a top-down approach to planning. This distinction may not be apparent to people within local government where, for some, the mandatory nature of the system is one of its most salient features. In Australia's three-tiered system of government, the state is not a single homogeneous entity. Tensions exist between spheres of government and between the centre and periphery. In Victoria these tensions include feelings amongst some in local government that State government fails to understand the local context and that it tends to treat local government as its administrative arm rather than as a duly elected separate sphere of government. Wills [21] argues that the State government's failure to consult with local government when developing the legislative amendments, and the concern that some in local government had that the changes were motivated by a desire to shift costs, created additional tensions.

In this environment the Act is open to at least two interpretations. The first is that it represents a genuine attempt to improve health planning at local government level. The second is that it is an attempt by State government to assert control over the process of planning. In the discussion paper preceding the Act's amendment HDV noted that local government were well placed to play an expanded role in public health because they were closer to the local community and had the necessary staff. However they noted that in two instances State government had a role to play: where there was insufficient local expertise – particularly in specialised functions; and where health problems extended beyond municipal boundaries. Additionally they recommended that "central government should retain oversight of local government in order to negate the undesirable effect on all Victorians of variability of public health enforcement by local councils" [22] (see page 20).

### The implementation

Although the Act was amended in 1988, implementation of Section 29(B) has been a lengthy process. A pilot study, with 11 councils, was undertaken in 1990 with the support of a project officer funded by HDV. During the following decade an increasing number of councils developed plans. This was done in an environment of uncertainty and tension. The election, in 1992, of the Kennett Coalition government introduced a period of tremendous change for local government. Driven by the prevailing ideology of 'small government', efficiency and market models the Coalition forced the amalgamation of many local governments (from over 200 to 78) and introduced a system of compulsory competitive tendering. Although amalgamations have long been a feature of the local government environment Australia wide, in Victoria the nature and scope of the 'reforms' was dramatic [14] (see page 99) and had long-term consequences. These changes created tension in the relationship between State and local government. They also stifled cooperation between local government as staff and operational units became wary about sharing information with counterparts in other local governments in the newly competitive tendering environment. In addition there was a significant impact on the public health workforce. Legge and Cox report that in the two years to 1997 there was an estimated 40% reduction in the number of environmental health officers within local government [19] (see page 11).

In 1999 a new State government was elected and this, perhaps, injected new enthusiasm into the implementation process. In 2001 the DHS and the Municipal Association of Victoria (MAV) released an MPHP planning framework in the form of the document *Environments for Health *[17]. This was intended as a guide for those within municipal councils responsible for writing the MPHP. This may have partially addressed some of the concerns that had been raised [19][20] regarding the skill capacity of the workforce within local government, to undertake strategic planning for public health. In 2004 a second document, *Leading the Way: Councils Creating Healthier Communities *[18], was released. Developed by VicHealth, DHS, and MAV, the document was designed to increase the understanding of elected councillors and local government senior management. It recognises, as the literature had highlighted, that senior level support is a critical factor in developing and implementing a strategic MPHP [19][20][23]. These two documents were supplemented by the *Good Practice Program*, through which from 2002 – 2004 the DHS funded 26 projects involving 39 local governments in activities that used *Environments for Health *to improve public health planning [24].

### The value of the MPHP framework

The significant variation in council approaches to planning, evident in the interview data, is a manifestation of the differences in councils' organisational capacity and culture. Many of those in local government would argue that financial resources are a critical component. There is, within local government, a measure of wariness about what is perceived as cost shifting by State and Federal government. A number of those who were interviewed discussed this with reference to the MPHP process. They saw the role and responsibilities of local government expand without any commensurate increase in funding. Clearly there is a relationship between levels of funding (whether directly from the State or made available internally) and a council's ability to develop and implement their MPHP. What is less obvious is the degree of correlation between councils' financial resources and their ability to deliver public health outcomes. Analysis of the interview data suggests that those councils who take a strategic approach to MPHP, who make reference to the social model of health, who have high levels of community involvement and who have formal processes in place for implementing and evaluating their plans are not, as a matter of course, the wealthier municipalities. Nor were those councils who demonstrated a narrow approach to public health planning or who lacked processes for implementation and evaluation necessarily the less well financially resourced. Other relevant factors include managerial support for the MPHP process (and public health programs and activities more generally), organisational culture, community expectations, and the skill and experience of those responsible for driving the process.

The persistence of differences in councils' approaches to planning also reflects that there is no shared or agreed understanding of the nature or level of the plans. Dever identifies three levels of planning: policy planning – what ought to be done, strategic planning – what can be done, and operational planning – what will be done [25]. He notes that "health planners must recognise the level of planning appropriate in a given situation" [25] (see page 43). It is not clear exactly what level of planning MPHPs involve, policy, strategic or operational, and, as evidenced by the interview data, local governments approach it in different ways. Smith suggests that because the requirement to produce a MPHP is contained in health legislation it is often seen, within local government, as the responsibility of the council's health unit or Environmental Health Officers (EHO). As a result it is perceived not as a strategic plan but as simply another function to be undertaken by the EHOs or health unit [20] (see pages 26–27). The report by McBride and Hulme recommends that those responsible for the development of the MPHP have "sufficient authority and legitimacy to effectively drive the public health process across the organisation" [23] (see page 41). In policy documents the framework is most often talked about as a strategic planning process [17,18]. However, it is not clear from these documents exactly what strategic planning is. For example, the DHS talks about "strategic local area planning" and a "strategic and integrated approach" without ever defining *strategic *[17] (see page 6). This distinction is important for public health where being able to anticipate and respond to emerging challenges is vital. It is *strategic *planning, rather than any other form, that constitutes that capacity. The fact that what is planned for is often overtaken by other demands diminishes the currency of the plans – particularly in the eyes of those directly involved in the process. Plans are often seen as best case scenarios not minimum goals to be achieved.

Regulation to encourage organisational behaviour change is a blunt instrument and certainly the MPHP framework is not enough in itself. However, where there are other factors encouraging effective planning, for example supportive management, the framework does act as a lever. Perhaps the most significant way in which it helps is as a platform for public health practitioners within local government – where it legitimates their activities. These people work within an environment of limited power, with multiple competing demands. Public health activities not already specifically described in legislation (that is activities other than immunisation, food sampling, premises inspection, and water inspection) are often not given a very high priority.

Health Department Victoria argued that it did not want to:

"restrict itself to receipt of an annual report consisting of a 'shopping list' of inspections made, food confiscated, food sampled etc but should instead, in conjunction with local government, develop a comprehensive public health plan against which local government output is measured" [22] (see page 11).

If this statement is used as a marker of State government's intention in introducing the policy, then the results have been mixed. Evidence from the interviews suggests that prior to the implementation of the framework, public health planning within local government, where it existed, generally did resemble the 'shopping list' model of inspections and samples. What the DHS is now presented with by local government is more sophisticated, more forward looking and more likely to make reference to the social model of health. The National Public Health Partnership notes that, with respect to proactive public health strategies, Victoria through MPHPs now has the most explicit provisions of any state [14] (see page 97). Although there is a continuum from very strong to weak MPHPs, there is a sense from the majority of those interviewed that even in local governments where the planning function is not as strong as would be desired, there have been improvements.

Publications such as *Environments for Health *and *Leading the Way*, and the *Good Practice Program *have made a positive difference to the planning process but these were not introduced until a decade after the legislation came into force. It may be some time before a judgement can be made about how successful they have been in promoting improved planning. The recently announced external evaluation of *Environments for Health *may answer some of these questions [26].

The current review of the Health Act 1958 signals the Department's ambition to move away from a focus on the process of planning, as an end in itself, towards implementation, or planning as a means to an end. Concern that there needs to be increased attention paid to the implementation of MPHPs is not new. As a result of his research in 1994 Smith notes that "not a lot of planning for implementation has been performed and that the task of developing the MPHP document was the immediate priority" [20] (see page 21). The current review poses several questions related specifically to the MPHP process:

"12. Should the new Act place a greater emphasis on implementing the MPHP and achieving its outcomes, rather than just developing a document, and if so, how could this be achieved?

13. Should the new Act require that municipal councils set out how they intend to fulfil their statutory functions in their MPHPs?" [27] (see page 20).

Submissions in response to the discussion document [27] are mixed. There is clear agreement with the proposal that MPHPs be aligned more closely with councils' other planning activities (for example the Municipal Strategic Statement). However there is not agreement regarding an increased focus on implementation. A number of submissions argue for a shift of emphasis towards implementation, with the City of Ballarat arguing that "the lack of sanctions has caused many councils to either ignore them or pay lip service" [28] (see page 2) (see also for example [29][30]). Other responses claim that an increased emphasis on implementation is unnecessary, with the City of Stonnington noting that "there is adequate emphasis on implementation of a MPHP in the current legislation. That this has not resulted in measurable outcomes highlights the reluctance of councils to be a competing service provider ... particularly when there is not a funding component to a MPHP" [31] (see page 3). The sections dealing with MPHP (29A and 29B) form only a small part of the Health Act and the review discussion document is not explicit regarding whether changes to these sections have been prompted by perceived failures in the existing arrangement or by a shift in the broader paradigm of public administration towards a greater emphasis on implementation and outcomes. It argues that shifting the emphasis towards implementation and outcomes would "make planning a more meaningful exercise" [27] (see page 19). The most recent public statements from the DHS suggest that they favour the integration of planning processes and increased support (such as implementation and evaluation tools) over prescriptive legislation to encourage a greater focus on implementation [33] (see page 30–31).

In addition to the current Health Act review, there are other indications that the State government intends making changes to reduce the number of plans required of local government in the near future. In a recent speech to local government representatives a State government Minister argued that "we need to cut the number of plans covering local government, integrate them better and ensure that planning cycles coincide" [34] (see page 9). However, as one of the conference participants noted, this would need to involve real changes in the way State and local government work together so that 35 medium-sized plans did not simply become three very large plans with no reduction in the administrative burden involved [15] (see page 6).

## Conclusion

Despite argument about the extent to which the planning process has improved, those interviewed generally agreed that there has been some improvement. A mandatory system of public health planning has not removed the significant variation in councils' approaches to public health planning. Such an expectation would be unrealistic. The planning process occurs within a wider context, and public health units with resources and focus have been best placed to employ the framework. Where units are weak their planning remains less than optimal. However, the system has at least impelled local government to address the question of planning for public health.

The criticism that the focus has been primarily on the process itself, rather than on the outcomes, seems a just one. The outputs of planning processes can be measured (the document itself) but planning outcomes in terms of achievements (that is improvements in population health) are a lot harder to measure. This is not a problem specific to local government, nor to this framework, but is a feature of public health planning generally. In the current review of the legislation, State government has clearly signalled that it would like to see some improvement in this although it is not clear how this might be achieved. Introducing a punitive element to the legislation, so that councils who failed to develop and implement a plan were in some way punished, would be counter productive. For any number of reasons, tensions exist in the relationship between State and local government. In such an environment, apparent 'poor' planning may be more than simply a matter of local government lacking the capacity to do 'good' planning. In some cases it may represent attempts by local government to resist what they see as another example of State government asserting control over their territory. Introducing a punitive element into the legislation would exacerbate the situation.

Victoria has had a system of mandated public health planning for over a decade. Despite criticisms of the system it is difficult to argue that mechanisms, other than legislative compliance, would be any more successful. In Victoria's case, where the local delivery system is very diverse, a legislative imperative has created, at the very least, a minimum standard for public health planning. The Victorian experience provides lessons for other jurisdictions as they consider how best to strengthen public health planning (see Table 1).

**Table 1:** Lessons from Victoria

▪ The process of legislative change needs to be truly consultative
▪ The type of plan and its scope should be explicit
▪ There needs to be shared understanding among stakeholders about the level of planning
▪ Winning the support of senior managers within local governments is vital
▪ Workforce capacity in planning is essential
▪ Integration with other planning activities, both within the organisation and at State level, is required
▪ There needs to be investment in implementation strategies

## Methods

Interviews were conducted with 10 people in nine local governments within one Department of Human Services (DHS) metropolitan region in Victoria, plus four additional interviews with other state based public health agencies. All of those interviewed were public servants. Within local government people were selected on the basis of their seniority and responsibility for public health, that is, people with highest level seniority whilst still retaining direct responsibility for public health services. The titles and responsibilities and levels of the positions varied as councils are often structured in different ways. Most commonly these people were titled Community Health or Health Services managers. In general they represent the middle layer of management within the council, reporting to a health/community services director or to the council's CEO/general manager.

The interviews were semi-structured. The intention was to repeat some core questions to allow for comparison between organisations, whilst retaining some flexibility to enable participants to raise issues that they regarded as important. The interview schedule was built around the broadest definition of infrastructure for public health developed by the National Public Health Partnership [10]. All interviews were audio recorded and transcribed verbatim. Thematic analysis was then employed to identify major themes emerging from the interviews.

The project had approval from La Trobe University's Human Ethics Committee (reference number 02–70)

## Competing interests

The author(s) declare that they have no competing interests.

## Authors' contributions

PB conducted the literature review, undertook data collection and analysis and drafted the paper. VL conceptualised the study, provided framing and structuring of the paper and commented on drafts. PS conceptualised the study and provided substantial comments on drafts. MW conceptualised the study and commented on drafts. TK provided key input into the framing of paper and current policy developments. KR provided comments on draft, including current policy developments.

All authors read and approved the final manuscript.
